# Hedinger Syndrome with Intraoperative Carcinoid Crisis — Understanding the Pathophysiology for a Successful Management

**DOI:** 10.21470/1678-9741-2020-0656

**Published:** 2022

**Authors:** Albert Franz Guerrero-Becerra, Juan Sebastián Frías-Ordoñez, Sergio A Higuera, Laura Patricia Gutiérrez-Soriano, Juan Camilo Giraldo

**Affiliations:** 1 Cardioinfantil Foundation, Cardiology Institute, Bogotá, Colombia.; 2 School of Medicine, Sabana University, Chía, Cundinamarca, Colombia.; 3 Rosario University, Bogotá, Colombia.

**Keywords:** Carcinoid Heart Disease, Tricuspid Valve Insufficiency, Carcinoid Tumor

## Abstract

Carcinoid tumors can be a cause for right heart valve disease, also known as Hedinger syndrome or carcinoid heart disease. Proper understanding of the pathophysiology is of the uttermost importance for adequate treatment of these patients, especially during heart surgery.

**Table:** Abbreviations, acronyms & symbols

IV	= Intravenous
MRI	= Magnetic resonance imaging
NYHA	= New York Heart Association

## INTRODUCTION

A 58-year-old female was admitted to the emergency room with increasing shortness of breath accompanied by gradually worsening edema of the lower limbs. She was on a New York Heart Association (NYHA) functional class III, and relevant physical examination findings included a grade III systolic murmur predominantly on the tricuspid auscultatory zone, significant edema of the lower limbs, and distended jugular veins. The electrocardiogram showed sinus rhythm with signs of right heart chambers overload.

The patient had a history of primary carcinoid disease of the ileum with hepatic and local nodal metastases. Given the extent of the disease, the primary tumor was deemed not suitable for surgical treatment and the patient was receiving medical treatment.

Considering her past medical history of carcinoid tumor, Hedinger syndrome was the most likely differential diagnosis. Other possible causes for right heart valve disease, such as infective endocarditis, or right heart failure, such as *cor pulmonale*, were much less likely given the absence of fever, no history of intravenous (IV) drugs use, and no history of chronic obstructive pulmonary disease.

**Fig. 1:**
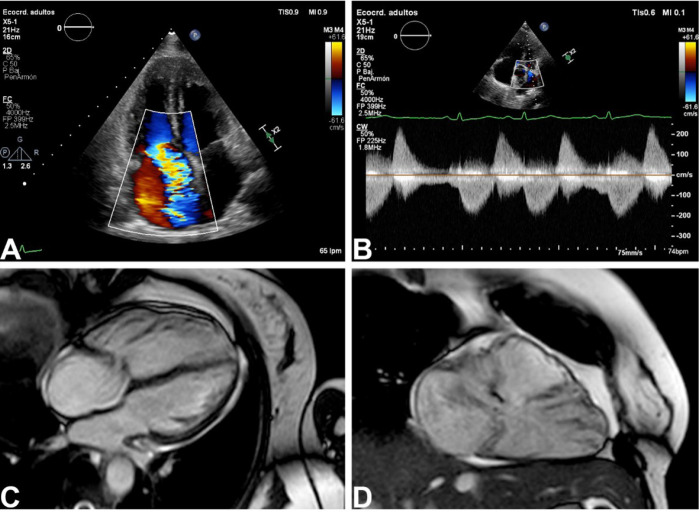
Echocardiography and cardiac magnetic resonance image (MRI) findings. On transthoracic echocardiography, the patient had severe tricuspid leaflet thickening causing severe regurgitation (A) and severe pulmonic regurgitation (B). On cardiac MRI, there is severe right ventricle dilation (C) and clear absence of valvar coaptation (C, D).

After management with diuretics and improvement of the fluid overload, the patient underwent transthoracic echocardiography showing typical findings of Hedinger syndrome. Cardiac magnetic resonance imaging (MRI) showed a severely dilated right ventricle, torrential tricuspid regurgitation, and severe pulmonary regurgitation (Figure 1). Her levels of 5-hydroxyindoleacetic acid demonstrated good medical treatment. Given these findings, surgical replacement of the tricuspid and pulmonary valves was decided by the Heart Team.

The patient received pre-surgical (150 mg, four times a day, 48 hours before surgery) and intraoperative (50 mcg bolus, followed by 50 mcg/h throughout) octreotide for diminishing the risk of carcinoid crisis. Despite proper treatment, she presented refractory hypotension before initiation of cardiopulmonary bypass; supplemental bolus of 50 mcg of octreotide was required with resolution of hypotension. Occasional episodes of transitory hypotension were treated with phenylephrine, and surgery was successfully completed.

**Fig. 2:**
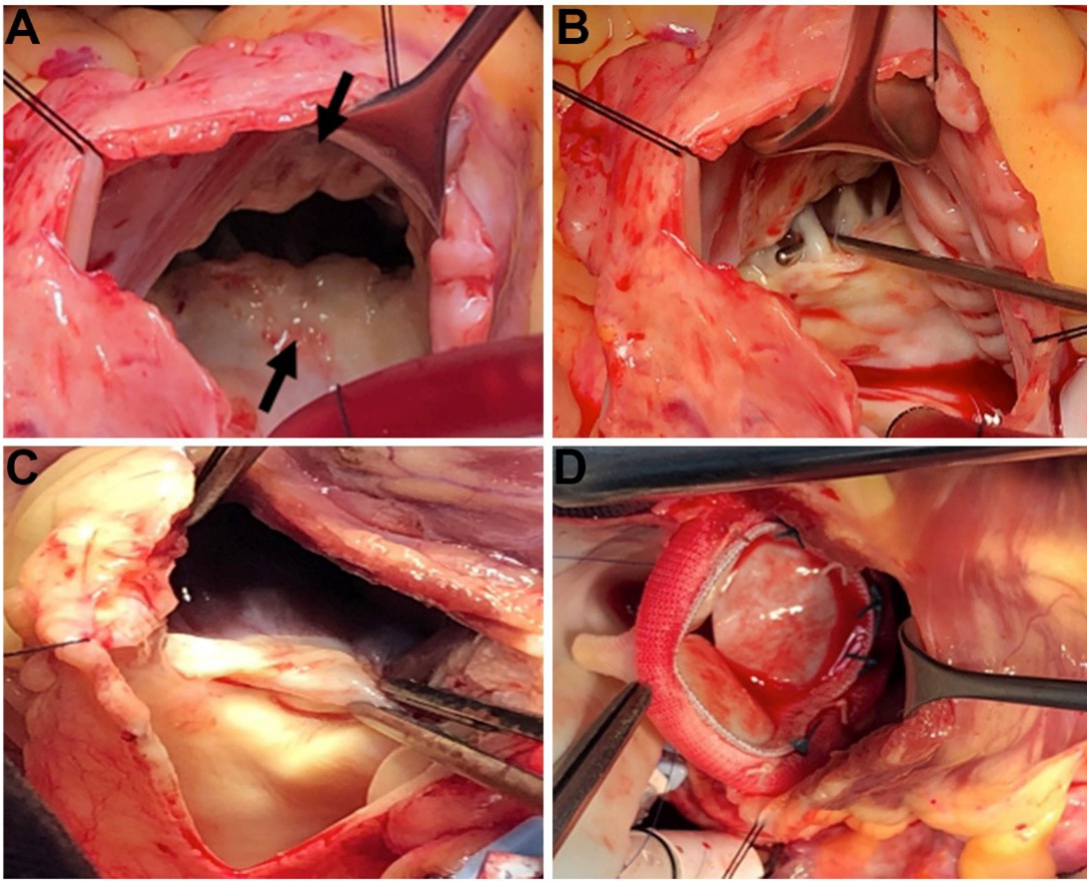
Surgical findings. A) Tricuspid valve with severe retraction of the anterior and septal leaflets (arrows). B) Severe retraction of the septal leaflet with thickening of the chordae tendineae. C) Severe retraction of the pulmonary valve. D) Bioprosthesis in the pulmonary position.

The patient underwent surgery through a median sternotomy, the heart was arrested after cardiopulmonary bypass was established with aortic and bicaval cannulation. Surgical findings showed absence of the tricuspid and pulmonary valves secondary to fibrosis (Figure 2). After right atriotomy, we implanted a bioprosthesis in the tricuspid position (Hancock Nº 31). Then we performed longitudinal pulmonary arteriotomy extending to the right ventricular outflow tract and placed a bioprosthesis (Hancock Nº 29) in the pulmonary position. Finally, we reconstructed the right ventricular outflow tract and the pulmonary artery with a bovine pericardium patch.

During the postoperative intensive care unit stay, she presented refractory hypotension, requiring IV octreotide infusion (100 mcg/h) with hemodynamic compensation and weaning from vasopressors. She was transferred to a general ward and then discharged home. After discharge, the patient has had a notorious improvement of symptoms. On her last clinical appointment, twelve months after surgery, she had a functional class NYHA I, with a transthoracic echocardiogram showing proper prosthetic valve function.

## DISCUSSION

Carcinoid tumors are neuroendocrine malignancies with an incidence of 1,2 - 2,1 cases/100,000 individuals. These malignancies, usually of the digestive tract, release substances derived from the metabolism of dietary tryptophan (serotonin, histamine, among others). These substances are responsible for the typical symptoms: secretory diarrhea, flushing, bronchospasm, and, in some patients, cardiac manifestations^[1]^.

Carcinoid heart disease, also known as Hedinger syndrome, is a rare cause of valvular heart disease, usually occurring in the setting of hepatic metastases. Patients present with right-sided valvular dysfunction; initial stages are well tolerated but progression to right heart failure is unavoidable without definitive treatment of the malignancy. Hedinger syndrome is associated with poor clinical outcomes, having a three-year survival rate compared with patients without heart compromise. Given the vasoactive capabilities of these substances, one of the deadliest complications are carcinoid crises. Carcinoid crises during cardiac surgery can severely complicate cardiac anesthesia and even cause intraoperative mortality^[2]^. Here we present a patient with Hedinger syndrome with an intraoperative carcinoid crisis and the management that allowed completion of surgery.

Typical findings of Hedinger syndrome include severe right-sided valve dysfunction with right ventricular dilation/dysfunction. The tricuspid and pulmonary valves have thickened leaflets that severely impair their motion and coaptation, causing severe regurgitation and right ventricular systolic dysfunction. Although transthoracic echocardiography can accurately assess tricuspid regurgitation, cardiac MRI may be required for evaluation of right ventricular systolic function, especially in cases with pulmonary valve dysfunction. Left-sided valve dysfunction is extremely rare (the pulmonary vascular bed usually metabolizes the released substances), and whenever present should always raise suspicion of pulmonary metastases^[3]^.

Despite medical management with diuretics, surgical treatment is the only definite treatment for heart failure in patients with severe valve dysfunction and right ventricular systolic dysfunction. Given the severe thickening of valve leaflets, valve repair is not feasible, and the overwhelming majority of patients undergo valve replacement. Bioprosthesis can present early dysfunction (mitigated with proper control of the malignancy), but usually do not require long-term anticoagulation and is therefore preferred. Balloon valvuloplasty of the pulmonary valve for patients of high surgical risk presenting with pulmonary stenosis can aid in palliation of symptoms, although failure of treatment is frequent^[4]^.

Carcinoid crises are defined as life-threatening hypotension caused by the release of vasoactive hormones from the malignancy, complicated by non-responsiveness to standard treatment with crystalloids and vasopressors. Patients with Hedinger syndrome undergoing cardiac surgery require preoperative treatment with somatostatin-analogs for inhibiting the release of vasoactive hormones. Intraoperative octreotide infusion is required and patients presenting with refractory hypotension may require additional IV bolus of octreotide. Failure to recognize the special anesthetic needs of these patients can further complicate surgery and result in mortality^[5]^.

## CONCLUSION

This clinical case exemplifies how, despite proper treatment, patients can present with carcinoid crises, which require a proper understanding of pathophysiology for adequate treatment. Failure to recognize this can lead to “usual” treatment of hypotension with vasopressors (*e.g*., norepinephrine), which can further deepen the carcinoid crisis, causing hemodynamic collapse and cardiac arrest. A multidisciplinary approach is required, preparing the team for state-of-the-art management and optimal outcomes.
